# Exposure to continuous political violence: rational and experiential thinking styles, coping styles and post traumatic stress symptoms

**DOI:** 10.3389/fpsyg.2023.1113608

**Published:** 2023-05-22

**Authors:** Liza Zvi, Keren Cohen-Louck

**Affiliations:** Department of Criminology, Ariel University, Ariel, Israel

**Keywords:** political violence, coping, thinking styles, post traumatic stress, PTSD

## Abstract

Cognitive style is considered an important determinant of individual behavior. The aim of the present study was to examine the relations between rational and experiential thinking styles, coping styles and Post Traumatic Stress (PTS) symptoms among civilians exposed to continuous and ongoing exposure to political violence. Three-hundred and thirty-two Israeli adult citizens living in the south region of Israel reported on their experiences of exposure to political violence as well as level of PTS, coping styles, and preference toward rational and experiential processing style. Results showed that low rational thinking was related with elevated PTS, both directly and indirectly through the mediation of high emotion-focused coping. The findings suggest that rational thinking may serve as a protective factor against stress related to chronic exposure to political violence; conversely, a preference for low rationality may be a risk factor.

## Introduction

Civilians who are exposed to continuous threats of political violence live in a constant state of stress. Such is the case of the citizens of south Israel over the past 20 years. The Israeli-Palestinian political conflict resulted in thousands of rockets and mortars fired from the Gaza Strip into south Israel, causing death, injury, and damage (Israel Ministry of Foreign Affairs, 2022). Arson attacks are also a threat and since March 2018, Palestinians are using explosive kites and balloons across the Gaza border, generating wide-scale fires and massive destruction of civilian property (Yarchi and Ayalon, 2020). Another source of concern for the people living in the area is Hamas’ terror tunnels. These tunnels, discovered during operation Protective Edge in 2014, are dug with the intention to infiltrate Israel and kill or kidnap Israeli soldiers or civilians (Zych, 2019). At times, the violence has escalated, and Israel reacts with retaliatory air strikes aimed at achieving deterrence and putting an end to the attacks (Zych, 2019; Loewenthal et al., 2021).

This ongoing violence between Israel and the Palestinians has taken its toll on civilians on both sides of the border. Studies indicated high prevalence of stressor related disorders (PTSD, Acute stress disorder), depression, generalized anxiety, and psychological distress (e.g., Ayer et al., 2015; Nuttman-Shwartz et al., 2015; Greene et al., 2018). Much of the research on the mental health impact of exposure to political violence has focused on the contribution of environmental and sociodemographic factors (e.g., Gelkopf et al., 2012; Greene et al., 2018; Shechory-Bitton and Cohen-Louck, 2020). However, individual differences may also play a role in the development of stress symptoms. For example, Besser and his colleagues have shown an association between levels of post-traumatic stress disorder (PTSD) and levels of anxious attachment (Besser and Neria, 2012), as well as optimism, self-esteem and hope (Besser et al., 2015). Big 5 traits of neuroticism, agreeableness, and conscientiousness were also associated with adaptation to stress and stress symptoms (e.g., Shmotkin and Keinan, 2011; Makkonen et al., 2020; Vasilopoulos and Brouard, 2020; Cohen-Louck and Zvi, 2022) and associations with personality structures were identified (Bachar et al., 2005; Besser et al., 2013). These studies indicate that personality factors may have a significant role in the proneness of individuals to experience Post Traumatic Stress (PTS) symptoms.

An emphasis on both environmental and personality factors in PTS research is in line with contemporary models of psychopathology seeing both factors as essential for maintaining mental health and for coping with and adaptation to stress (e.g., Besser and Neria, 2012; Noser et al., 2014). The diathesis stress model proposes that exposure to stress caused by life experiences interacts with individual differences to produce stress related health outcomes, suggesting that individuals with high levels of psychological vulnerabilities are at a higher risk for developing PTSD (McKeever and Huff, 2003). Cognitive vulnerability has been given considerable theoretical and empirical attention, with cognitive models positing that mental processes such as interpretation, attention, and memory mediate the relation between environmental events and emotional responses (Riskind and Alloy, 2006; Elwood et al., 2009). For example, such models anticipate that a person’s core assumptions and beliefs (e.g., the world is benign) may pose a risk factor for developing PTSD, and may change following traumatic experiences (e.g., the world is unsafe and unjust). A core assumption is that perceptual and information processes play a key role in understanding how personality operates following exposure to adversities (Robinson and Wilkowski, 2015).

## Rational and experiential thinking styles and PTS symptoms

The Cognitive Experiential Self Theory (CEST; Epstein, 1985) provides a framework for how people process information and arrive at decisions. CEST offers a dual-process model of information, comprised of rational and experiential pathways of information processing. The first pathway occurs consciously, and is effortful, logical, analytical, and reason oriented. The second is preconscious, involves rapid automatic processing, uses heuristics, and is highly affect-oriented. The degree to which individuals rely on either pathway is based on individual trait differences and situational demands (Epstein, 1985, 1990, 2003; Denes-Raj and Epstein, 1994).

A preference for using a rational thinking style has been associated with several measures related to good adjustment, including self-esteem, action orientation, and favorable views about the self and the world. It was also inversely related to a variety of measures related to poor adjustment, including anxiety, neuroticism, depression, alcohol abuse, overgeneralization, and stress (Epstein et al., 1996; Pacini et al., 1998; Pacini and Epstein, 1999). A preference for experiential processing has also shown several beneficial associations, including emotional expression extraversion and positive interpersonal relationships. However, it has also been associated with naïve optimism, superstitious beliefs, and stereotypical thinking (Epstein et al., 1996; Pacini and Epstein, 1999), as well as making biased decisions (Kahneman and Frederick, 2002; Shiloh et al., 2002; Gunnell and Ceci, 2010). On the contrary, a rational thinking style has been associated with normative statistical responses and judgments (Shiloh et al., 2002; Ayal et al., 2011).

To the best of our knowledge, there are no studies on the relation between rational and experiential information processing styles and PTS symptomatology. The aim of the present study was to evaluate whether the way in which information is processed (rationally and experientially) is related to PTS symptomatology due to continuous traumatic stress, among the citizens of south Israel exposed to political violence. Since rational thinking is associated with effective action to solve problems and with measures of good adjustment, we anticipated that it will be associated with low levels of PTS symptoms. On the other hand, reliance on affect-related cognitions and the use of heuristics (i.e., using the experiential system) may be associated with high levels of PTS symptoms. Therefore, we hypothesized that:

*H1*: Rational thinking style will be negatively related and experiential thinking style will be positively related with PTS symptoms.

## Rational and experiential thinking styles and coping styles

A second aim of the present study was to assess the relations between the two thinking styles and the strategies used for coping with the adversity of living amid continuous political violence. The two basic coping styles in facing political violence are problem- and emotion-focused (Cohen-Louck and Ben-David, 2017; Cohen-Louck and Levy, 2020). The first addresses the problem, whereas the second deals with the emotional and psychological outcomes of the problem (Lazarus and Folkman, 1984; Chesney et al., 2006; Folkman, 2013). Studies on coping with political violence have shown that problem-focused active coping strategies, like searching for suspicious objects and identifying suspects, protect against distress. In contrast, emotion-focused coping strategies like giving up, self-blame, dissociation, denial, and self-distraction increase the likelihood of experiencing stress and PTS symptoms (Gil, 2005; Braun-Lewensohn et al., 2009; Nuttman-Shwartz and Dekel, 2009).

The two ways of coping seem to correspond to the two thinking styles. The experiential system is highly affect-oriented, operating by the principles that govern automatic, associative learning, and solves problems in adaptation by automatically reacting according to a person’s reinforcement history. In contrast, the rational system employs deliberative thinking and solves problems by adhering to logical principles and evaluation of evidence (Norris and Epstein, 2011). Thus, thinking rationally may be related with problem-focused coping and thinking experientially may be related with emotion-focused coping. There are no studies, to the best of our knowledge, that examined the association between thinking style and coping style with political violence. However, previous research indicates inverse relations between rationality and emotional problems such as depression and anxiety, suggesting a negative link between rational thinking and emotion-focused coping. Based on the above, we hypothesized that:

*H2*: Problem-focused coping will be negatively related with PTS symptoms and emotion-focused coping will be positively related with PTS symptoms.

*H3*: Rational thinking style will be positively related with problem-focused coping, and negatively related with emotion-focused coping. Experiential thinking style will be positively related with emotion-focused coping.

Finally, we aimed at assessing a model for the relations between thinking styles, coping styles, and PTS. The literature indicates that problem-and emotion-focused coping are associated with PTS symptoms. The present study suggests associations between these coping styles and thinking styles. Therefore, we hypothesized that:

*H4*: Coping styles will mediate the relations between thinking styles and PTS.

A better understanding of the relations between thinking styles, coping styles, and PTS symptoms can be useful in preventive and therapeutic work with civilians that are coping with continuous political violence.

## Method

The present study is part of a research project designed to study factors associated with mental health consequences caused by exposure to continuous political violence among citizens living in the south region of Israel.

### Participants

Three hundred Israeli adult citizens participated in the current study. All lived in the south region and were exposed to long-term political violence. There were 198 (66.0%) females and 102 (34.0%) males, between the ages of 18 and 59 years (*M* = 25.80, SD = 7.84). They have been living in the area for up to 56 years (*M* = 16.33, SD = 9.73). Most were single (*N* = 233, 77.7%), and others were married (*N* = 59, 19.7%), with about 16% who had children (*N* = 49, 16.3%). Most were Israeli born (*N* = 272, 90.7%) as well. They were secular (*N* = 126, 42.0%), partly religious (*N* = 79, 26.3%), or religious (*N* = 95, 31.7%), and had a high school (*N* = 179, 59.7%) or an academic (*N* = 116, 38.7%) education.

### Instruments

#### Exposure to political violence

The participants reported their experience with incidents of political violence. Three questions were posed, based on previous research (Shechory-Bitton, 2013; Shechory-Bitton and Cohen-Louck, 2018). Based on these three questions, three variables of exposure to political violence were computed. The first two variables, in accordance with the definitions stated by Gelkopf et al. (2012), were: 1. Personal exposure to political violence: (0) no exposure, (1) heard or saw a missile fall, (2) physically injured; 2. Family/friend exposure to political violence: (0) no exposure, (1) family member or friend heard or saw a missile fall, (2) family member or friend was physically injured. The third variable was named prolonged exposure and pertained to the number of periods of time the participant perceived themselves to have been exposed to political violence, ranging from zero (0) to five (5).

#### Rational experiential inventory

To assess individual use of rational (deliberative) and experiential (intuitive) processing modes, Epstein et al. (1996) created the Rational Experiential Inventory (REI). Shortened versions of the REI are also in use (Pacini and Epstein, 1999), including a 24-item version (e.g., Ayal et al., 2011, 2012), which was used in the present study. The REI and its shortened versions are considered valid and acceptable measures (e.g., Björklund and Bäckström, 2008; Sánchez et al., 2012; Monacis et al., 2016; Shirzadifard et al., 2018). The REI includes two unipolar scales, ranking participants on the two dimensions of thinking styles: rational analytic (e.g., I do not think it is a good idea to rely on one’s intuition for important decisions; *α* = 0.80) and experiential-intuitive (e.g., I often go by my instincts when deciding on a course of action; *α* = 0.85). Scores were composed of the mean of the items so that higher scores represent higher deliberative or intuitive cognitive styles (range 1–5).

#### Coping

Carver et al.’s COPE 30 item Hebrew version scale was used to measure coping (Carver et al., 1989; Ben-Zur and Zeidner, 1995). The participants were asked to report the extent to which they used different coping options in dealing with the ongoing threats of the missile attacks on a 4-point scale ranging from 0 (*not at all*) to 3 (*to a great extent*). Higher scores represent greater use of each strategy. The internal consistency indices were: Problem-focused: *α* = 0.88 and emotion-focused: *α* = 0.79.

#### Posttraumatic stress (PTS) symptoms

The PTSD Symptom Levels (PSL) questionnaire (Gil et al., 2015, 2016) was used to measure posttraumatic stress symptoms (PTS). The PSL consists of 20 self-report items that correspond to the 20 DSM5 diagnostic criteria for PTSD. The participants were asked to rate each item on a 4-point scale of severity ranging from 0 (*not at all*) to 3 (*severely*). Higher scores reflect greater PTS. Due to positive skewness values, these variables were log transformed. The internal consistency indices were: *α* = 0.95 for the total score, *α* = 0.90 for Intrusion, *r* = 0.77 (*p* < 0.001) for Avoidance, *α* = 0.91 for Negative alterations, and *α* = 0.86 for Arousal.

### Procedure

Participants were recruited through Facebook postings during December 2018, inviting civilians from the south region of Israel facing the threat of political violence to participate in a study on responses to political violence. Data collection lasted until December 2019, a period during which the southern region of Israel had been the target of 584 missile attacks. Interested individuals were directed to an online questionnaire where they were provided with information about the nature of the study and their rights as research participants, including the right to anonymity and the right to end their participation at any time. Upon providing their informed consent to participate in the study, the participants were directed to complete the online questionnaire (see the Instruments section). The study was approved by the Ethics committee of the university.

### Data analysis

Data were analyzed with SPSS version 28. Internal consistencies were calculated, and variables were composed with item means or sums. The PTS variable was positively skewed and was thus log transformed. The study variables were described with means and standard deviations, and Pearson correlations were calculated between them. Pearson correlations and independent t-tests were calculated between PTS and the demographic and background characteristics, to identify background variables that needed to be controlled for. The hypotheses were examined with a path analysis, using AMOS version 28. Chi square, *NFI*, *NNFI*, *CFI*, and *RMSEA* were used as measures of model fit. Family/friend exposure to political violence, prolonged exposure to political violence, duration of living in the area, and religiosity were controlled for in the analysis. Control variables were allowed to correlate among themselves, and so were the independent variables, and the mediators. Mediation effects were assessed within the path analysis, with 5,000 bootstrap samples and 95% confidence interval.

## Results

Hearing or seeing a missile fall was reported by most participants (*N* = 279, 93.0%), and four participants (1.3%) reported being injured. All participants were exposed to political violence, and in most cases – personally (*N* = 283, 94.3%) (others were exposed to it through family members or friends). Further, in most cases a family member or a friend were reported to hearing or seeing a missile fall (*N* = 171, 57.0%), or being injured (*N* = 107, 35.7%). Participants reported experiencing an average of 2.86 periods of political violence (*SD* = 1.68, range 0–5). About 11% of them were classified in the clinical range of PTSD (*N* = 33, 11.0%).

Low variance was noted for personal exposure and was thus excluded from the analyses. Family/friend exposure to terror was dichotomously defined: (1) a family member or a friend was injured, (0) a family member or a friend heard or saw a missile fall, or none. Prolonged exposure was used as a continuous variable as it ranged from 0 to 5, and did not deviate from normal distribution (skewness = −0.20, SE = 0.14).

Several relationships were found between the background variables and PTS. PTS was higher when a family member or a friend was injured (*M* = 15.43, SD = 14.30) than when they heard or saw a missile fall (*M* = 11.89, SD = 11.80) [*t*(200.68) = 2.08, *p* = 0.039]. It was positively, yet weakly, related with prolonged exposure (*r* = 0.14, *p* = 0.017), and to duration of living in the area (*r* = 0.17, *p* = 0.004). Further, higher levels of PTS were found for partly religious and religious participants (*M* = 14.64, SD = 13.53) than for secular participants (*M* = 11.10, SD = 11.55) [*t*(298) = 2.35, *p* = 0.020]. Other background variables were unrelated with PTS. The hypotheses were thus examined while controlling for family/friend exposure, prolonged exposure, duration of living in the area, and religiosity (correlations among these variables ranged between *r* = 0.01 *p* = 0.995 and *r* = 0.15 *p* = 0.010).

Table 1 presents the distribution of the study variables and intercorrelations among them. Results show a mean PTS of about 13 out of about 60. Means for coping strategies were moderate and means for thinking styles were moderate-high. Low to moderate intercorrelations were found among the study variables. A lower use of the rational thinking style, and a greater use of both problem-focused and emotion-focused coping, were related with higher PTS symptoms. The rational and experiential thinking styles were positively interrelated, and the problem-and emotion-focused coping strategies were positively interrelated as well. Higher rational thinking style was related with lower emotion-focused coping.

**Table 1:** Means, standard deviations and intercorrelations for the study variables (*N* = 300).

	*M* (SD)	1	2	3	4	5	6	7
1.Total PTS (0–59)	13.15 (12.84)	1						
2. Family/friend exposure (0–1)	0.36 (0.48)	0.13*	1					
3. Prolonged exposure (0–5)	2.86 (1.68)	0.13*	0.15*	1				
4. Rational thinking style (1–5)	3.59 (0.60)	−0.24***	0.12*	0.02	1			
5. experiential thinking style (1–5)	3.76 (0.58)	−0.03	0.07	0.07	0.19**	1		
6. Problem-focused coping (0–3)	1.55 (0.69)	0.28***	0.07	0.04	0.01	0.13*	1	
7. Emotion-focused coping (0–3)	1.23 (0.47)	0.51***	0.13*	0.12*	−0.20***	−0.07	0.57***	1

*p < 0.05, **p < 0.01, ***p < 0.001.

**Figure 1:**
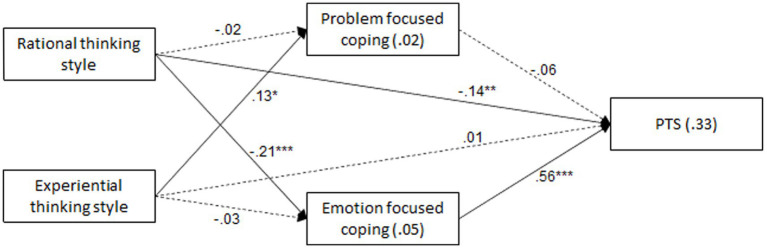
Path analysis for PTS, thinking styles, and coping strategies. *p < 0.05, **p < 0.01, ***p < 0.001. *R*^2^ – percent of explained variance-values within rectangles. β – standardized regression coefficients-values above arrows. Solid lines – significant relationships; dashed lines – non-significant relationships.

The hypotheses were examined with a path analysis, using AMOS ver 28. Family/friend exposure, prolonged exposure, duration of living in the area, and religiosity were controlled for. Control variables were allowed to correlate among themselves, and so were the independent variables, and the mediators. Continuous variables were standardized. The model was found to fit the data: *χ*^2^(16) = 17.46, *p* = 0.356, NFI = 0.948, NNFI = 0.989, CFI = 0.995, RMSEA = 0.018. Table 2 and Figure 1 present the relations between PTS, thinking styles, and coping strategies.

**Table 2:** Path analysis for PTS, thinking styles, and coping strategies (*N* = 300).

Dependent variable (*R*^2^)	Independent variable	*B*	SE	*p*
Problem-focused coping (0.02)	Rational thinking style	−0.02	0.06	0.743
	experiential thinking style	0.14	0.06	0.022
Emotion-focused coping (0.05)	Rational thinking style	−0.21	0.06	<0.001
	experiential thinking style	−0.03	0.06	0.603
Total PTS (0.33)	Rational thinking style	−0.05	0.02	0.005
	experiential thinking style	0.01	0.02	0.839
	Problem-focused coping	−0.02	0.02	0.273
	Emotion-focused coping	0.20	0.02	<0.001

Results show significant direct relationships. Higher rational thinking style was related with lower emotion-focused coping, and higher experiential thinking style was related with higher problem-focused coping. Further, higher rational thinking style and lower emotion-focused coping were related with lower PTS. One indirect relationship was found significant—relating rational thinking style with PTS (Indirect effect = −0.042, SE = 0.011, *p* < 0.001, 95% CI: −0.065, −0.022). Higher rational thinking style was related with lower emotion-focused coping, which was then related with lower PTS.

Analysis of the effect size of the indirect effect, according to Preacher and Kelley (2011) revealed that the unstandardized effect size was −0.12, showing that PTS was expected to decrease by 0.12 units per unit change in rational thinking style indirectly through emotion-focused coping. The partially standardized effect size was −0.33, showing that PTS was expected to decrease by 0.33 standard deviations for every one-unit increase in rational thinking style (on its 5 point scale) indirectly through emotion-focused coping. The standardized effect size was −0.36, indicating that PTS decreased by 0.36 standard deviations for every 1 SD increase in rational thinking style indirectly through emotion-focused coping.

That is, rational thinking style was negatively related with PTS, both directly and indirectly, through the mediation of emotion-focused coping. Experiential thinking style was positively related with problem-focused coping, yet both were unrelated with PTS.

## Discussion

The aim of the present study was to evaluate whether the way in which information is processed (rationally and experientially) is related to the way of coping (emotion-and problem-focused) with chronic exposure to political violence and the level of PTS among civilians living in the south region of Israel.

The findings point to the significance of the rational thinking style, showing that low rational thinking is related with elevated PTS, both directly and indirectly through the mediation of high emotion-focused coping. These results are in line with previous research demonstrating a positive link between rational thinking style and measures related to good adjustment, as well as a negative link between rational thinking style and measures related to poor adjustment, including anxiety, neuroticism, depression, alcohol abuse, overgeneralization, and stress (Epstein et al., 1996; Pacini et al., 1998; Pacini and Epstein, 1999; Norris and Epstein, 2011). The present findings support the existing evidence on the link between rational thinking style and adjustment and add to them. They mainly suggest that a preference for rational thinking may serve as a protective factor against stress related to chronic exposure to political violence; conversely, a preference for low rationality may be a risk factor.

It was further hypothesized that reliance on affect-related cognitions and the use of heuristics (i.e., using the experiential system) will be related with using emotion-focused coping and with high levels of PTS symptoms. However, the findings do not support this. A possible account for these results may be related to a dominance of the rational system over the experiential system. According to Epstein’s model, when people respond to an emotionally significant event, they automatically begin to process the encountered information through the experiential system. The subsequent processing of the rational system may be biased by the automatic responses of the experiential system; however, the logical rational system can also correct the experiential system. People often reflect on their initial spontaneous thoughts and impulses and suppress or substitute those considered inappropriate (Epstein, 2012). According to Ayal et al. (2012), deliberative but not intuitive thinking is the crucial predictor of behavior. Initial intuitive preferences may or may not be manifested depending on the strength of the deliberative process. Thus, among individuals characterized by high rational thinking, the rational thinking process adjusts or overrides the initial intuitive preferences, directing behavior towards a rational solution. The present findings may support this reasoning, as they show that only rational thinking, but not experiential thinking, is associated with PTS symptoms.

As expected, emotion-focused coping was associated with elevated PTS. Previous research has indicated that emotion-focused coping increases the likelihood of experiencing prolonged stress and post traumatic symptoms (Braun-Lewensohn et al., 2010; Weinberg et al., 2014). Emotion-focused coping also emerged as a mediator between rational thinking style and PTS, showing the expected association between low rationality and greater use of maladaptive coping strategies, which in turn are related with greater PTS symptoms. The expectations regarding problem-focused coping were not supported. The findings indicate that problem-focused coping was not related with better adaptation (i.e., lower levels of PTS). The literature generally emphasized the superiority of problem-focused coping (e.g., Weinberg et al., 2014), however there are also studies with contradictory findings, showing that elevated use of both emotion-and problem-focused coping strategies is related to stress symptoms (e.g., Cohen-Louck and Ben-David, 2017; Cohen-Louck and Levy, 2020), as well as studies showing that using problem-focused strategies may only be adaptive in the short-term after exposure to traumatic events, yet ineffective in the long-term (e.g., Kanninen et al., 2002; Schaufeli and Bakker, 2004). It was suggested that efforts to solve practical aspects related to traumas, characteristic of problem-focused coping, are beneficial when traumatic experiences are still fresh. However, in the long run such efforts may be counterproductive in the absence of an available solution (Kanninen et al., 2002; Schaufeli and Bakker, 2004). It was further suggested that problem-focused coping may be related with elevated anxiety levels as it is associated with paying more attention to the stressor, its source, and solutions, yet reemphasizing its uncontrollable aspects (Forsythe and Compas, 1987; Gidron et al., 1999). The focus of the present study was on PTS resulting from exposure to political violence which constitutes a continuous situation involving various stressors, many of which are uncontrollable. It is possible that under these conditions, problem-focused coping strategies may be ineffective.

The prediction concerning the relations between thinking styles and coping styles was based on the assumption that problem-focused coping is an indicator of good adjustment. However, as described, the findings suggest a more complex association of problem-focused coping and stress reactions in the context of continuous political violence. If problem-focused coping does not constitute a rational solution, then an association between rational thinking style and problem-focused coping should not be expected. While rationality was unrelated with problem-focused coping, it was related with emotion-focused coping, showing the expected association between a high preference for rational thinking and lower use of maladaptive coping strategies, which may subsequently lead to lower PTS.

The present study points to the importance of rational thinking in coping with continuous political violence. The findings show that high rationality is more related to better adaptation and better adjustment than low rationality. According to Epstein, the experiential system has both adaptive and maladaptive aspects, making its relation to adjustment weaker (Epstein et al., 1996). The present results suggest that the rational thinking style is a better indicator of adjustment than the experiential thinking style. They also support previous research pointing to a possible primacy of the rational thinking over the experiential thinking (Ayal et al., 2012; Zvi and Shechory-Bitton, 2022).

The results add to what is already known about the significance of cognitive factors (e.g., dysfunctional appraisals of traumatic events, negative beliefs about self and others, low perceived control over recovery) to PTSD development, maintenance, and treatment (e.g., Dunmore et al., 2001; Kindt et al., 2008; Marks et al., 2018).

## Conclusion

This study contributes to the body of literature by presenting a model describing a relationship between thinking style, coping style, and PTS symptoms. According to our model, rational thinking may serve as a protective factor against stress related to chronic exposure to political violence and emotion-focused coping mediates the relationship between rational thinking style and stress symptoms. To the best of our knowledge, this study is the first to examine the association between thinking style and PTS symptomatology and the first to examine the association between thinking style and coping style with political violence.

There are considerable individual differences in the ways in which individuals generally adapt to the environment, and particularly in adaptation to stress and adversity. While research has mostly focused on the effects of various sociodemographic factors (e.g., socioeconomic status, gender, age, marital status) as well as environmental factors (e.g., social support, proximity to the border) on PTS (Gelkopf et al., 2012; Shechory-Bitton and Cohen-Louck, 2020), there is a need for deeper knowledge on the role of individual differences in adaptation to and coping with stress and trauma (Besser et al., 2015; Skalski et al., 2021). The present study aimed to fill this gap by examining the contribution of thinking styles.

Our findings underline issues significant to therapeutic practice with victims of continuous political violence. Practitioners responsible for public health should provide designated treatments which will focus on promoting rational thinking patterns that advance adaptation and help reduce the use of maladaptive coping strategies. Additionally, efforts should be made to advance personal strength and well-being (e.g., emotional stability and optimism).

The present study is not without limitations. Firstly, the present study focused on assessing specific stress responses, in the context of continuous and ongoing threat. It is possible that different characteristics of threat and coping will produce different outcomes. Future research on factors such as the duration of stress (continuous vs. related to a one-off trauma), time of coping (short-term vs. long-term after exposure), and level of perceived controllability will allow for better understanding of the relations between thinking styles, coping styles and PTS; specifically, to test whether different conditions will yield different associations between thinking styles, coping styles and PTS. Further research on the role of individual differences is needed. For example, information processing may be affected by personality (Zvi and Elaad, 2016; Elaad and Zvi, 2019). It may be informative to look at such personality effects with respects to using rational and experiential thinking styles and PTS symptoms. Integrating the effects of sociodemographic and environmental factors on PTS susceptibility with the effects of personality will allow for a more compressive understanding of risk factors. Furthermore, ways of coping with stress and adversity may be culturally dependent. Future studies should examine the associations between thinking styles, coping styles and PTS among people from different racial and ethnic groups, socioeconomic status, and status of residency in the country. For example, many Israelis are immigrants who came to live in the country within the Law of Return (Shechory and Ben-David, 2010). Being a stressful life event that requires long adaptation, immigration might add to the difficulties of coping with political violence. Thus, it may be informative to examine whether immigrants who suffer continuous political violence show different associations between thinking styles, coping and PTS, in comparison to native born Israeli citizens. Future studies should also examine the relationship between thinking styles, coping style and PTS symptoms among the Palestinians, as well as among the Arab Israelis who are affected by the political violence (Shechory Bitton and Silawi, 2019).

Another limitation of the present study concerns its cross-sectional nature. Further research is necessary to detect causal pathways between thinking style, coping styles, and PTS symptoms. Furthermore, the external validity of the findings may be somewhat limited because the sample was a convenience sample and since this study is based on self-reports, the results represent participants’ subjective assessment of their thinking style, ways of coping, and levels of traumatic stress. Finally, future studies may consider examining this study’s assumption within different cultural and cross-cultural contexts.

## Data availability statement

The raw data supporting the conclusions of this article will be made available by the authors, without undue reservation.

## Ethics statement

The studies involving human participants were reviewed and approved by the study was approved by the Ethics committee of the university. The patients/participants provided their written informed consent to participate in this study.

## Author contributions

The contribution of LZ and KC-L to this study’s conception, design, questionnaire development and writing and reviewing of the manuscript was equal. LZ led the organizing and analyzing of the database. Both authors contributed to the article and approved the submitted version.

## Conflict of interest

The authors declare that the research was conducted in the absence of any commercial or financial relationships that could be construed as a potential conflict of interest.

## Publisher’s note

All claims expressed in this article are solely those of the authors and do not necessarily represent those of their affiliated organizations, or those of the publisher, the editors and the reviewers. Any product that may be evaluated in this article, or claim that may be made by its manufacturer, is not guaranteed or endorsed by the publisher.
